# Computational and experimental mechanical performance of a new everolimus-eluting stent purpose-built for left main interventions

**DOI:** 10.1038/s41598-021-87908-2

**Published:** 2021-04-22

**Authors:** Saurabhi Samant, Wei Wu, Shijia Zhao, Behram Khan, Mohammadali Sharzehee, Anastasios Panagopoulos, Janaki Makadia, Timothy Mickley, Andrew Bicek, Dennis Boismier, Yoshinobu Murasato, Yiannis S. Chatzizisis

**Affiliations:** 1 Cardiovascular Biology and Biomechanics Laboratory, Cardiovascular Division, University of Nebraska Medical Center, 982265 Nebraska Medical Center, Omaha, NE 68198 USA; 2 Boston Scientific, Maple Grove, MN USA; 3 Department of Cardiology, National Hospital Organization, Kyushu Medical Center, Fukuoka, Japan

**Keywords:** Cardiac device therapy, Interventional cardiology, Biomedical engineering

## Abstract

Left main (LM) coronary artery bifurcation stenting is a challenging topic due to the distinct anatomy and wall structure of LM. In this work, we investigated computationally and experimentally the mechanical performance of a novel everolimus-eluting stent (SYNERGY MEGATRON) purpose-built for interventions to large proximal coronary segments, including LM. MEGATRON stent has been purposefully designed to sustain its structural integrity at higher expansion diameters and to provide optimal lumen coverage. Four patient-specific LM geometries were 3D reconstructed and stented computationally with finite element analysis in a well-validated computational stent simulation platform under different homogeneous and heterogeneous plaque conditions. Four different everolimus-eluting stent designs (9-peak prototype MEGATRON, 10-peak prototype MEGATRON, 12-peak MEGATRON, and SYNERGY) were deployed computationally in all bifurcation geometries at three different diameters (i.e., 3.5, 4.5, and 5.0 mm). The stent designs were also expanded experimentally from 3.5 to 5.0 mm (blind analysis). Stent morphometric and biomechanical indices were calculated in the computational and experimental studies. In the computational studies the 12-peak MEGATRON exhibited significantly greater expansion, better scaffolding, smaller vessel prolapse, and greater radial strength (expressed as normalized hoop force) than the 9-peak MEGATRON, 10-peak MEGATRON, or SYNERGY (*p* < 0.05). Larger stent expansion diameters had significantly better radial strength and worse scaffolding than smaller stent diameters (*p* < 0.001). Computational stenting showed comparable scaffolding and radial strength with experimental stenting. 12-peak MEGATRON exhibited better mechanical performance than the 9-peak MEGATRON, 10-peak MEGATRON, or SYNERGY. Patient-specific computational LM stenting simulations can accurately reproduce experimental stent testing, providing an attractive framework for cost- and time-effective stent research and development.

## Introduction

Drug-eluting stents (DES) have dominated the field of percutaneous coronary interventions, with the current generation stents offering thinner struts, better deliverability and good safety and efficacy profiles^1^. Restenosis is still a significant problem with drug-eluting stents, particularly in bifurcation lesions^2,3^, despite the reduction in neointimal hyperplasia associated with their use^4^. The stent design, stenting technique and the associated biomechanical environment are major factors leading to bifurcation stent restenosis^5,6^. This becomes more pertinent to the left main (LM) bifurcation interventions, given the distinct anatomical features of LM. Specially designed stents with improved expansion capabilities and lumen scaffolding would have a theoretical advantage in LM interventions^7,8^.

Computational simulations using finite element analysis (FEA) has evolved to a powerful tool to study stent geometry (e.g. number of links, strut thickness, strut cell size), which directly impacts stent performance (e.g. radial and longitudinal stent strength, stent deliverability, lumen scaffolding, plaque prolapse, side branch access), and subsequent clinical outcomes^7,9^. Patient-specific computational stent simulations require meshing, assignment of boundary and loading conditions, and validation against bench experiments^10–12^. Once validated, computational simulations can be used for stent testing and development, reducing the development time and associated costs^13,14^. To date several studies have used FEA to study the structural and biomechanical properties of stents (Table 1). The majority of these studies have been performed in idealized vessel models, whereas in others there was no lumen environment at all for the stent expansion^15–22^. A limited number of studies used patient-specific coronary artery models, however, they did not take into account the true wall thickness and composition, thereby, reducing the ability of the analyses to produce realistic results^23,24^. Our study advances the current state-of-the-art by performing a comprehensive analysis of different stent designs expanded within a patient-specific bifurcation environment that incorporates realistic vessel wall thickness and material properties.

**Table 1:** Computational stent modeling studies.

References	Aim	Stent designs	Vessel model	Performance metric
Samant et al. (current study)	Study computationally and experimentally the performance of novel Everolimus-eluting stent designs	MEGATRON 9-, 10- and 12-peak designs and SYNERGY (Boston Scientific)	Patient-specific left main bifurcations with homogeneous wall (spectrum of very soft to very stiff) and patient-specific heterogeneous wall	Stent expansion Vessel scaffolding Vessel prolapse Stent-artery ratio Normalized hoop force/ Radial strength
Bobel et al.^12^	Assess the performance of biodegradable stents	MultiLink, Absorb (Abbott) and Igaki–Tamai (Kyoto Medical Planning Co.)	Parallel network viscoelastic material model	Radial stent strength Stent Flexibility Longitudinal stent resistance
Ragkousis et al.^17^	Assess the longitudinal integrity of first and second-generation drug eluting stents in a patient-specific coronary artery segment	Promus element, Promus element modified, (Boston Scientific) Xience (Abbott), and Cypher (Johnson and Johnson Co.)	Patient-specific straight coronary artery segment with homogeneous wall	Longitudinal stent deformation Stent malapposition
Roy et al.^25^	Computational performance of various commercially available stent designs	Palmaz Schatz, Cypher (Cordis, J & J), S670, Driver (Medtronic,), Taxus Express, Element (Boston Scientific)	Free stent expansion (without vessel)	Von Misses stresses
Chiastra et al.^24^	Computational fluid dynamic studies in stented coronary models	Xience Prime (Abbott) and Endeavor Resolute (Medtronic)	Patient-specific coronary bifurcation lumen with idealized homogeneous wall	Computational fluid dynamic studies
Boyle et al.^26^	Long term restenosis outcomes of different stent designs on mechanobiological model of arterial tissue	MultiLink (Abbott), Palmaz (Johnson and Johnson Co.), and Inflow (Inflow Dynamics)	Idealized vessel models	Neointimal tissue growth
Conway et al.^18^	Assess the performance of different designs in straight and curved vessels	Cypher (Johnson and Johnson Co.) and MultiLink (Abbott)	Idealized straight and curved arterial models with homogeneous wall	Vessel recoil after stenting Vessel scaffolding von Mises stresses
Grogan et al.^19^	Study the performance of bio-absorbable stents	Generic and alloy-specific stent designs of magnesium, iron, steel and cobalt-chromium	Free stent expansion (without vessel)	Radial stent strength Stent recoil Stent flexibility Longitudinal stent resistance von Mises stresses Principal logarithmic stent strain
Mortier et al.^20^	Evaluate the mechanical behavior of different stent platforms	Integrity (Medtronic), Veriflex (Boston Scientific), MultiLink 8 (Abbott), Multi-Link Vision (Abbott Vascular), Pro-Kinetic Energy (Biotronik), and Promus Element (Boston Scientific)	Idealized non-bifurcated vessel models with homogeneous wall	Stent malapposition Vessel wall stresses
Wu et al.^21^	Optimize the shape of biodegradable magnesium alloy stents	Four different variation of magnesium alloy stent compared to the existing Magic (Biotronik)	Idealized straight vessel with homogeneous wall	Vessel scaffolding Stent recoil Maximum principal stress and strain
Gijsen et al.^23^	Study stent deployment in patient-specific coronary artery segment	Bx velocity (Cordis, Johnson and Johnson)	Patient-specific straight coronary artery segment with homogeneous wall	Luminal and stent stresses
Migliavacca et al.^22^	Assess the mechanical stent performance of different stent designs	Palmaz-Schatz, MultiLink Tetra (Guidant) and Carbostent (Sorin Biomedica)	Free stent expansion (without vessel)	Radial and longitudinal stent recoil Stent foreshortening
Etave et al.^27^	Determine mechanical characteristics of different stent designs	Palmaz-Schatz (Johnson and Johnson Co.) and Freedom (Global Therapeutics Inc.)	Free stent expansion (without vessel)	Elastic recoil Longitudinal and radial stent resistance Vessel Scaffolding Stent flexibility Stress maps

In this work, we studied computationally and experimentally the mechanical performance of a new everolimus-eluting stent (SYNERGY MEGATRON, Boston Scientific Inc., Galway, Ireland), purposefully designed for large proximal vessels, including LM bifurcations. The MEGATRON stent geometry has been optimized for high radial strength and overexpansion to 6.0 mm, while maintaining vessel scaffolding to address the specific needs of these large vessels. In optimizing the final MEGATRON design, three developmental designs were explored that varied with respect to the number of peaks (9-peak, 10-peak, and 12-peak), but maintained identical overexpansion capability to 6.0 mm. The comparative MEGATRON stent designs are referred to herein as 9-peak, 10-peak, and 12-peak, with the 12-peak being the final MEGATRON commercial design. The n = 3 everolimus-eluting MEGATRON stent designs were compared computationally and experimentally with each other, as well as against the existing everolimus-eluting SYNERGY stent. For the computational study, we compared the n = 4 stent designs using different plaque materials and expansion diameters in patient-specific LM bifurcations using a well validated patient-specific computational stent simulation platform. Our study revealed the importance of patient-relevant computational stent simulations in stent testing and development.

## Methods

### Computational studies

#### Patient data

We retrospectively selected n = 4 LM bifurcation geometries with significant disease from the patient database of Kyushu Medical Center, Fukuoka, Japan. The use of these geometries was approved by the ethics committee of Kyushu Medical Center (Institutional Review Board #20C035). All methods were carried out in accordance with relevant guidelines and regulations, and informed consent was obtained from all subjects. All n = 4 cases underwent coronary angiography at multiple angiographic planes and intracoronary imaging of left anterior descending and left circumflex artery or intermediate branch with OCT before the percutaneous coronary intervention.

#### 3D reconstruction of LM bifurcations

**Figure 1:**
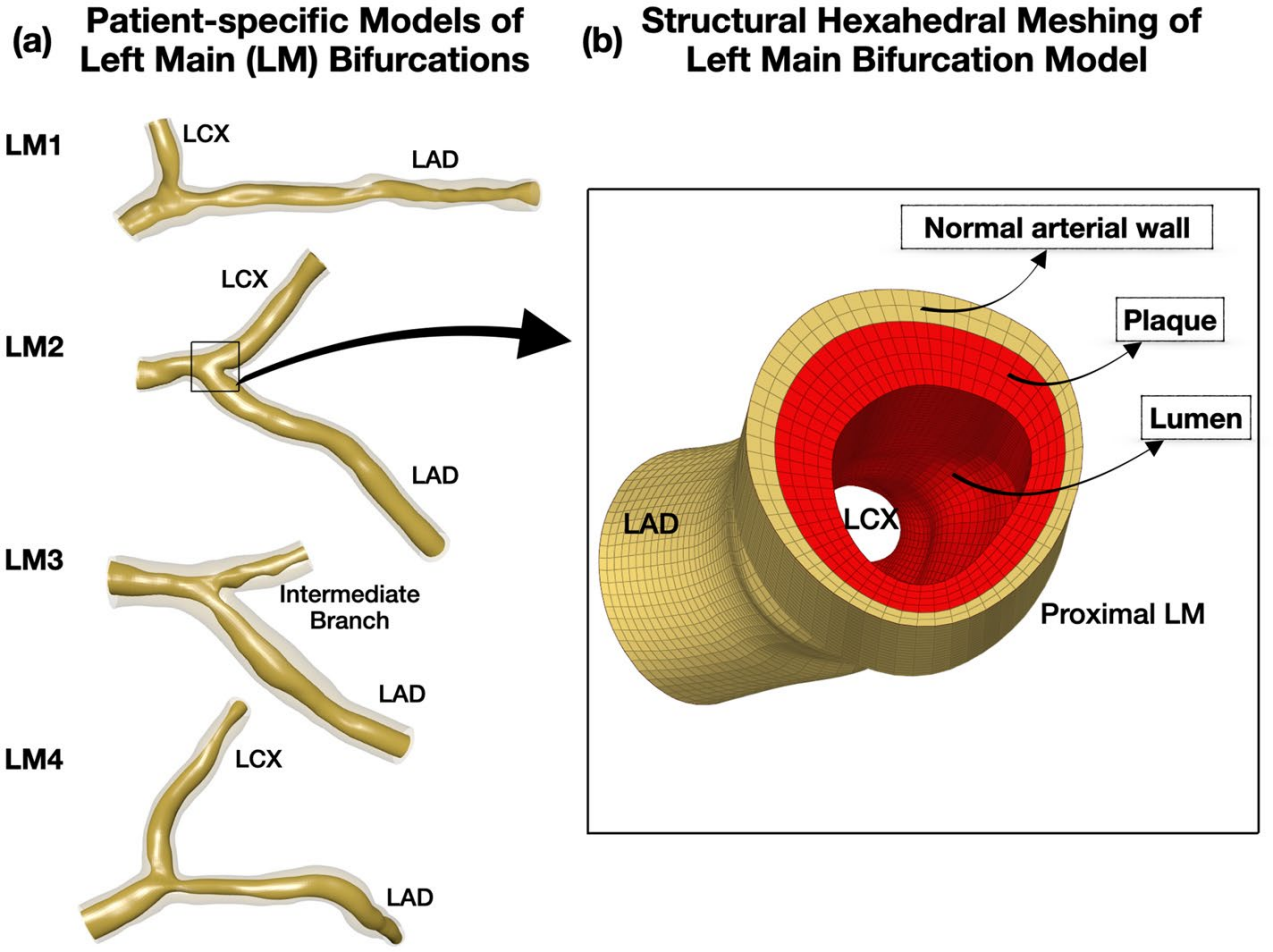
3D reconstructed patient-specific left main (LM) bifurcation geometries. (**a**) 3D reconstructed lumen and wall of n = 4 patient-specific LM geometries using angiography and OCT imaging, (**b**) Magnification of the structured hexahedral mesh of a 3D reconstructed bifurcation. Note the normal wall (yellow) and plaque (red); LAD: Left anterior descending, LCX: Left circumflex artery.

We 3D reconstructed the LM bifurcations by fusing the pre-procedural angiography and OCT using a well-validated technique^28^. Briefly, we used two angiographic planes, at least 30 degrees apart, to generate the bifurcation centerline. We segmented the lumen and wall of left anterior descending and left circumflex artery or intermediate branch from OCT images. The segmented lumen and wall contours were positioned on the 3D reconstructed bifurcation centerline, generating a realistic 3D representation of the bifurcation anatomy (Rhinoceros 3D, Robert McNeel and Associates, WA, USA, Fig. 1a).

#### Stent designs

**Figure 2:**
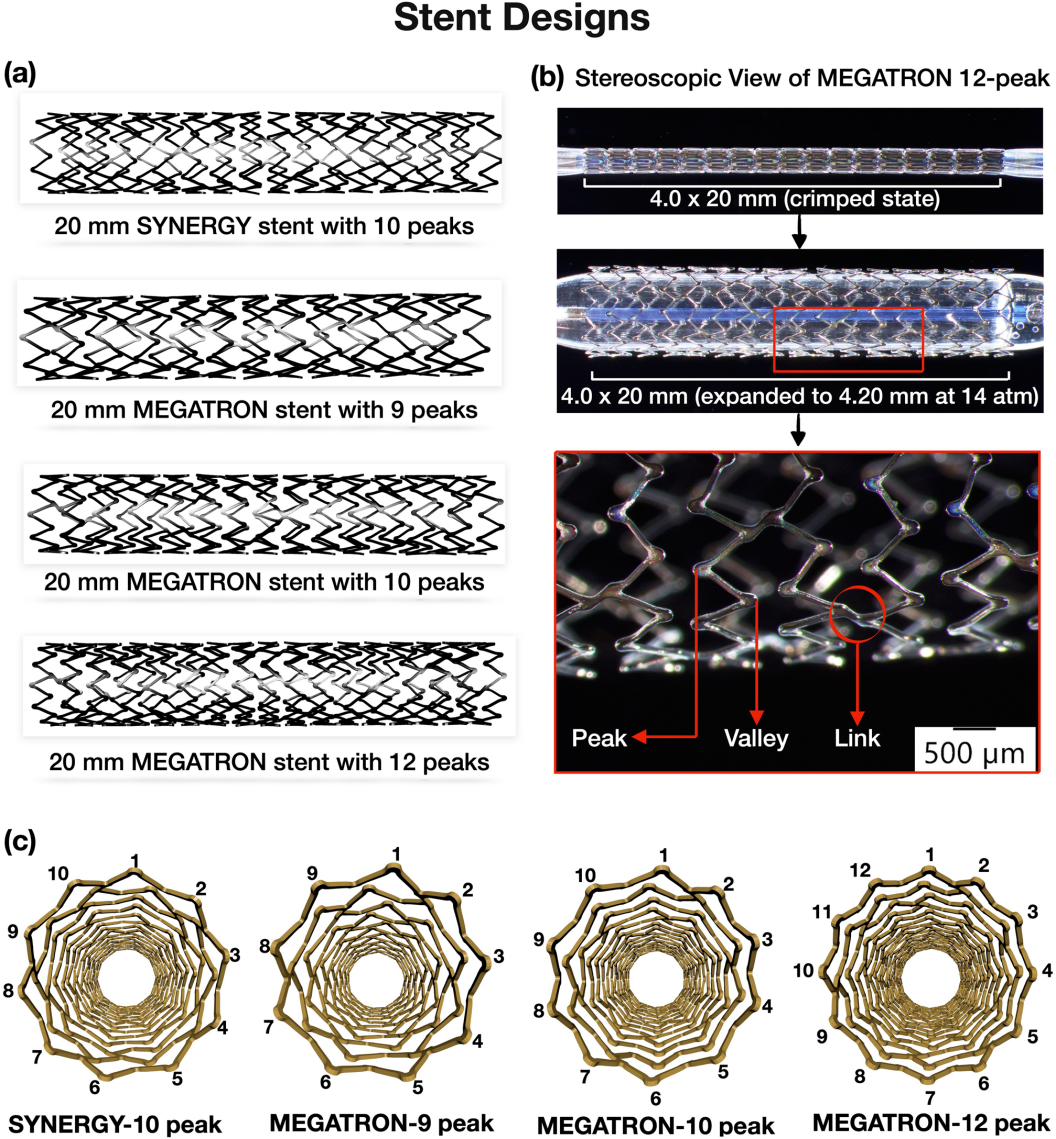
Stent designs. (**a**) Everolimus-eluting stent designs used in the computational and experimental testing: 9-peak, 10-peak, 12-peak MEGATRON and SYNERGY, (**b**) Stereoscopic view of crimped and expanded 12-peak MEGATRON stent. Note the stent struts peaks, valleys and links, (**c**) Cross-sectional configuration of the n = 4 stents.

We obtained the computer-aided design models of the platinum chromium everolimus-eluting stent models (i.e. n = 3 MEGATRON designs, and n = 1 SYNERGY design) from the manufacturer (Boston Scientific, Galway, Ireland). The stent length was chosen according to the length of stenosis. The n = 4 stent designs were, 20 mm in length, inner diameter 0.84 mm, and outer diameter 1.02 mm at crimped state and were different with respect to the number of peak-to-valleys circumferentially and axially connected links. Details about the stent designs are provided in Fig. 2 and Table 2.

**Table 2:** Characteristics of different stent designs.

Stent design	Peaks	Crimped length (mm)	Links	Strut thickness (µm)
MEGATRON	9	19.74	36	89
	10	20.62	28	89
	12	20.05	50	89
SYNERGY	10	20.33	36	81

#### Meshing

The stents were meshed in HyperMesh (Altair Engineering, Michigan, United States) using hexahedral elements. The multi-fold stent balloons were generated in Grasshopper (plugin to Rhinoceros 3D 6.0) at their crimped state and meshed with quadrilateral finite-membrane-strain elements. The vessel wall was meshed using hexahedral elements with 0.35 mm global mesh size. Mesh convergence study with different element sizes, ranging from 0.35 to 0.50 mm showed negligible impact on stent expansion (less than 1% relative difference). We used 1–2 element layers to represent normal arterial wall and 2–4 element layers to represent plaque (Fig. 1b).

#### Material properties

##### Homogeneous plaque materials

**Figure 3:**
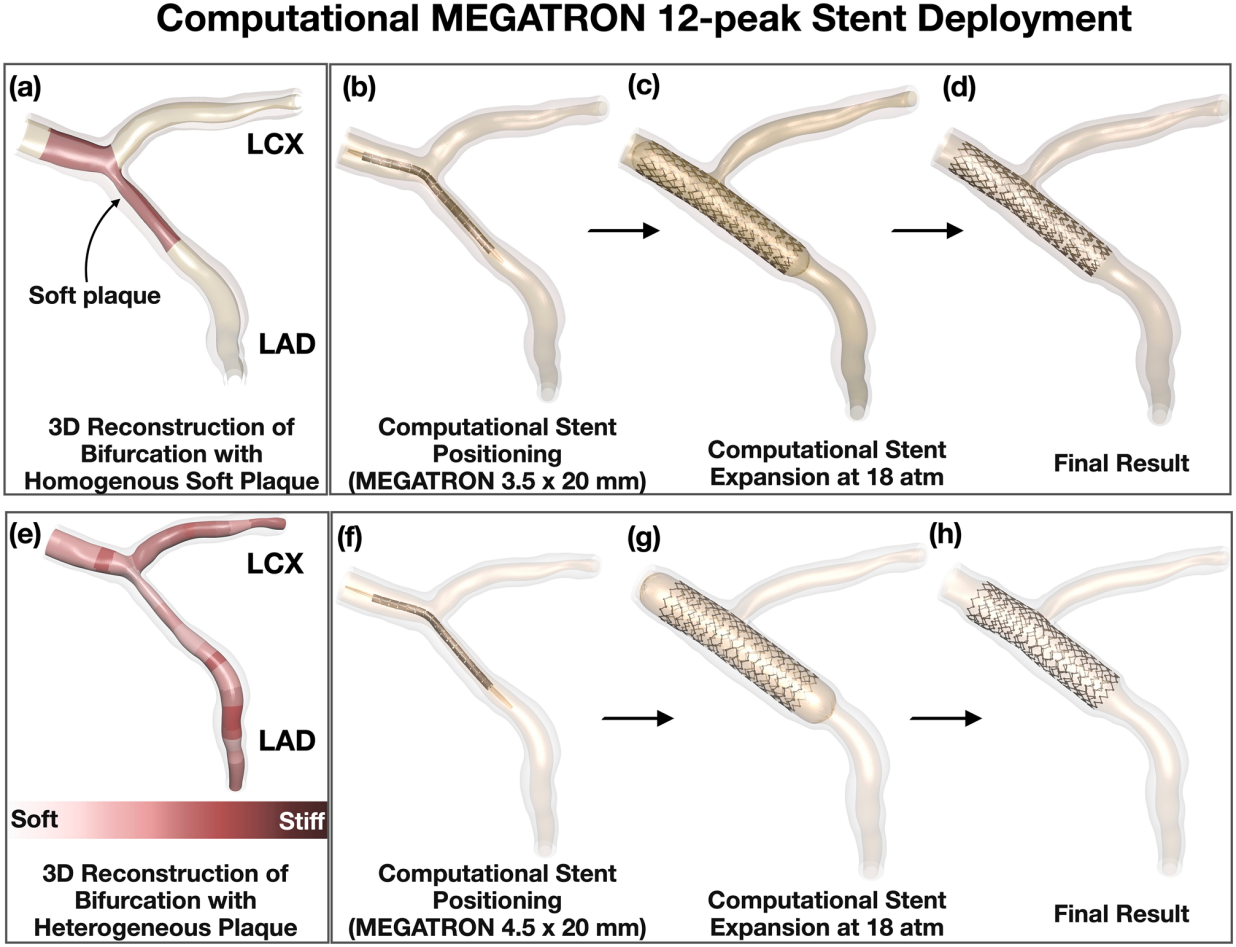
Representative examples of computational MEGATRON 12-peak stent expansion. (**a**) 3D reconstruction of LM bifurcation lumen and wall by angiography and optical coherence tomography and assignment of homogeneous plaque material properties (soft in this example), (**b**–**d**) Computational MEGATRON stent positioning, expansion, and final result, (**e**) Assignment of heterogeneous (patient-specific) plaque stiffness to the 3D reconstructed LM bifurcation. Note the various zones of patient-specific differential plaque stiffness across the length and circumference of the lumen, (**f**–**h**) Computational MEGATRON stent positioning, expansion, and final result; LAD: Left anterior descending, LCX: Left circumflex artery.

In each LM bifurcation, we assigned normal artery wall material in the outer 0.1 to 0.25 mm of the wall. The normal artery wall material was modeled using the sixth-order reduced polynomial constitutive equation, which characterizes the isotropic hyper-elastic mechanical behavior^20^. The strain energy density for the polynomial hyperelastic constitutive equation was expressed as $U = \sum\limits_{i + j = 1}^{N} {C_{ij} (I_{1} - 3)^{i} (I_{2} - 3)^{j} }$, where $C_{ij}$ are material coefficients determined from the experimental data, while $I_{1}$ and $I_{2}$ are the first and second invariant of the Cauchy-Green tensor in terms of principal stretch ratios $\lambda_{i}$ as $I_{1} = \lambda_{1}^{2} + \lambda_{2}^{2} + \lambda_{3}^{2}$_,_ $I_{2} = \lambda_{1}^{ - 2} + \lambda_{2}^{ - 2} + \lambda_{3}^{ - 2}$. The coefficients for normal arterial layer were obtained by fitting the equation to the experimental data^29,30^. In areas with plaque, we assigned 5 different homogeneous plaque material properties, i.e. very soft (lipid only), soft, neutral, stiff and very stiff (calcium only; Figs. 1b and 3a). The reduced polynomial strain energy density constitutive equation was used to fit the uniaxial tensile test data^31^. Plaque plasticity was initiated at 34% strain^32^. Four Platinum Chromium alloy (Pt–Cr) stent designs, modeled with the Von Mises-Hill plasticity model^33^ and semi-compliant balloons with elastic modulus of 900 MPa^34^ were computationally implanted in each homogeneous plaque material environment. The material coefficients for the normal arterial wall, plaque and Pt-Cr alloy stents are listed in Supplemental Table 1.

##### Heterogeneous (patient-specific) plaque materials

In addition to performing computational simulations with homogeneous plaque materials, we assigned heterogeneous plaque composition based on OCT. To account for the circumferential and longitudinal plaque heterogeneity, we calculated the following plaque morphometric parameters in every 10th OCT frame: plaque thickness, plaque eccentricity, lipid thickness and arc, calcium thickness and arc, fibrosis thickness and arc, fibrous cap thickness, and presence of necrotic core. In case of significant plaque transition between two adjacent 10th OCT frames, additional OCT frames were analyzed. The arterial wall was discretized into zones comprised of OCT frames with similar plaque morphometric characteristics. Based on these plaque characteristics, each zone was assigned a global stiffness category ranging from soft (lipid predominant) to stiff (calcium predominant; Fig. 3e). The global stiffness categories and the corresponding coefficients for plaque and normal arterial wall are summarized in Supplemental Table 1.

#### Computational stenting simulations

Computational stent expansion was carried out through a multi-step, quasi-static finite element analysis using the central difference method in Abaqus /Explicit solver (Dassault Systems Simulia Corp, Rhode Island, United States). For all analysis, the ratio of kinetic energy to the total internal energy was maintained below 5% and target time increments were set as 5 × 10^–8^ for inflation and 1 × 10^–7^ for deflation (adjusted via mass scaling) to obtain fast results while avoiding dynamic effects. In all patient-specific LM bifurcations, the stents were computationally deployed from the non-significantly diseased (< 50% plaque burden) part of LM to the non-significantly diseased part of left anterior descending artery (cross-over stenting; Figs. 3b–d and 3f–h). The stents mounted on to the crimped balloons were positioned across the left anterior descending artery, following the lumen centerline. The ends of vessel model were fixed and friction coefficient of 0.2 was applied to all contacting surfaces. The normal arterial wall and plaque had shared nodes at their interface, with no relative motion. All stents were computationally expanded at 18 atmospheres with semi-complaint balloons. The proximal stent expansion was sized to the proximal vessel reference (i.e. proximal LM), whose mean lumen diameter did not exceed 5.0 mm in any of the study cases.

### Computational stent performance metrics

#### Stent expansion

The mean inner stent diameter (MSD) was compared between the stent designs at different expansion diameters and plaque types. The difference in stent expansion between 9-peak and 12-peak MEGATRON stent was expressed as percentage difference using 9-peak stent as reference.

#### Vessel scaffolding

**Figure 4:**
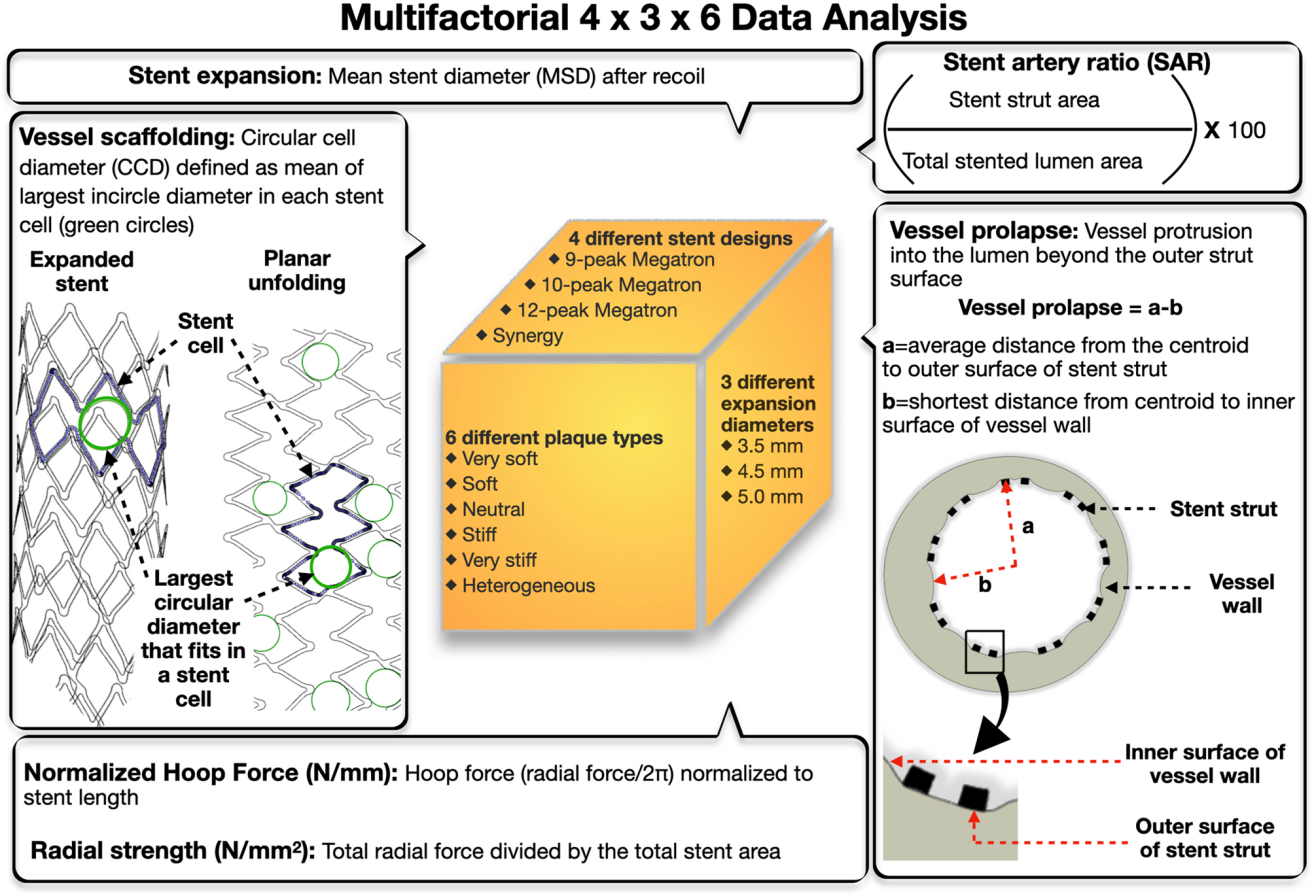
Design of computational analysis and definition of computational parameters. In our computational analyses, we factored 4 different stent designs, 3 stent expansion diameters and 6 plaque types in all n = 4 LM bifurcations. For each scenario, we calculated stent morphometric and biomechanical parameters i.e. stent expansion, vessel scaffolding, vessel prolapse, stent artery ratio, normalized hoop force, and radial strength.

After computational stent expansion, vessel scaffolding was assessed by the circular cell diameter (CCD), which was calculated as the average of the largest circles that could be inscribed in each stent cell after stent expansion (Fig. 4). CCD represented the vessel coverage by the stent, such that the smaller the CCD, the denser the strut pattern and the better the vessel scaffolding. Vessel scaffolding was calculated in stents expanded against different plaque materials.

#### Vessel prolapse

Vessel prolapse was measured as the radial distance from the centroid of the lumen to the maximum wall protrusion subtracted by the average radial distance from the lumen centroid to the outer strut surfaces (Fig. 4). The vessel prolapse was calculated with the stent expanding against normal arterial wall (without plaque material assignment).

#### Stent artery ratio

The stent-to-artery ratio represented the part of the lumen covered by stent struts. It was expressed as percentage of the stent strut outer surface area divided by the total lumen surface area in the stented part. Stent-to-artery ratio was calculated for all n = 3 MEGATRON stent designs with different expansion diameters in homogeneous neutral and heterogeneous plaque.

#### Normalized hoop force

Normalized hoop force was calculated by expanding the stents against homogeneous neutral plaque and heterogeneous plaque. Normalized hoop force was calculated as the hoop force normalized by the nominal stent length (N/mm). Hoop force was defined as radial force/2π^35^. Radial force was measured computationally as the sum of all outward radial forces exerted by the stent on the lumen wall. In this analysis, the normalized hoop force was used as an indirect measure of radial strength. To make the computational hoop force calculations comparable to the experimentally calculated hoop force (see “Normalized Hoop Force” section), we computationally simulated the radial crimping process. The 12-peak MEGATRON was computationally expanded to 4.5 mm (inner diameter), then we applied radial crimping using multiple rigid plates to achieve a compressed diameter of 3.5 mm (inner diameter). The maximum hoop force by computational analysis was 7.7 N and by experimental analysis was 7.6 N (Supplemental Fig. 1).

In addition to the normalized hoop force, we calculated directly the radial strength (N/mm^2^) of stents expanded at 4.5 mm against heterogeneous plaque. The radial strength was calculated as the total radial force divided by the total stent area.

### Experimental bench studies

The scaffolding and normalized hoop force of the n = 4 stent designs were assessed experimentally by expanding the stents against soft hydrogel material and placing in a radial crimping machine, respectively. The experimental studies were performed in Boston Scientific Inc. (Maple Grove, MN, USA), independently from the computational studies by a different group of researchers, who were blind to the computational analysis findings.

#### Scaffolding

Scaffolding was measured by an optical 360° scan of the stent geometry at each diameter of interest. The output of this scan allowed accurate measurements of the stent cell geometry. CCD was the measure used for assessment of scaffolding, as described in “Vessel Scaffolding” section.

#### Normalized hoop force

The hoop force of the deployed stent was measured using a 12-element radial force gauge (Machine Solutions Incorporated, Flagstaff, AZ, USA; Supplemental Fig. 2), which applied uniform radial pressure on the stent until the stent achieved a pre-determined (15%) reduction in diameter. A 15% reduction in stent diameter was selected in order to capture the onset of permanent, non-recoverable, compressive diameter change. The maximum compressive hoop force required to achieve the prescribed reduction in diameter was normalized by the measured nominal stent length to generate a normalized hoop force value (N/mm).

### Statistical analysis

GraphPad Prism 8.0 (GraphPad Inc., San Diego, CA, USA) was used to perform all the statistical analysis. Continuous variables were expressed as mean with standard error of mean (SEM). The normality of the data distribution was assessed with the Kolmogorov–Smirnov test. Group comparisons were performed using analysis of variance and Friedman’s tests with Bonferroni’s and Dunn’s tests, respectively, to correct for multiple comparisons. *P* < 0.05 was considered as the level of statistical significance.

## Results

**Figure 5:**
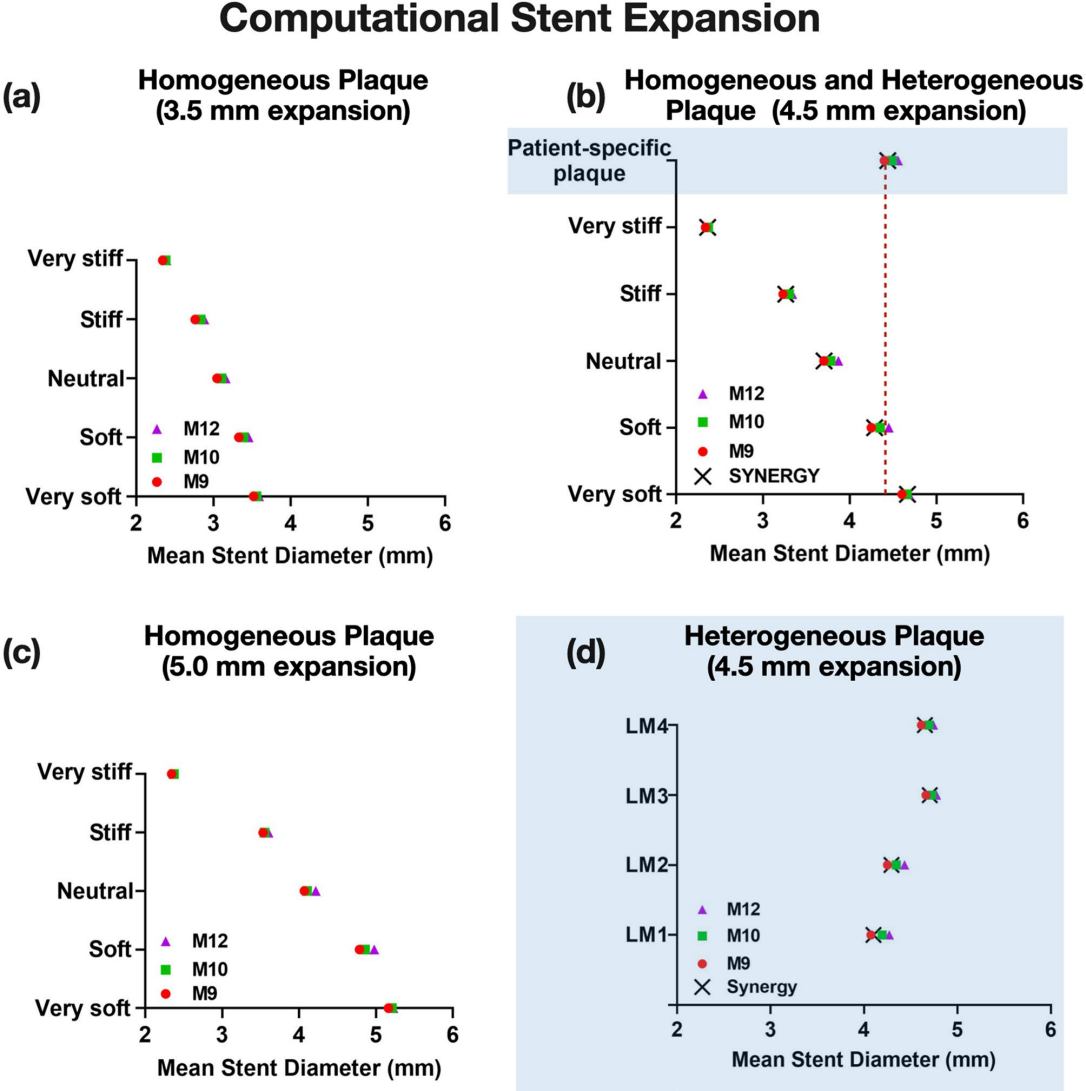
Computational stent expansion across different stent designs, diameters and plaque materials. Note the differential effect of plaque material and stent design on expansion of (**a**) 3.5 mm stents, (**b**) 4.5 mm stents, (**c**) 5.0 mm stents in homogeneous plaque environment. (**d**) The same pattern was seen when the stent designs were expanded within heterogeneous patient-specific plaques (shaded areas of graph) for all n = 4 left main (LM) geometries; M12: MEGATRON 12-peak, M10: MEGATRON 10-peak, M9: MEGATRON 9-peak, LM: Left Main.

**Figure 6:**
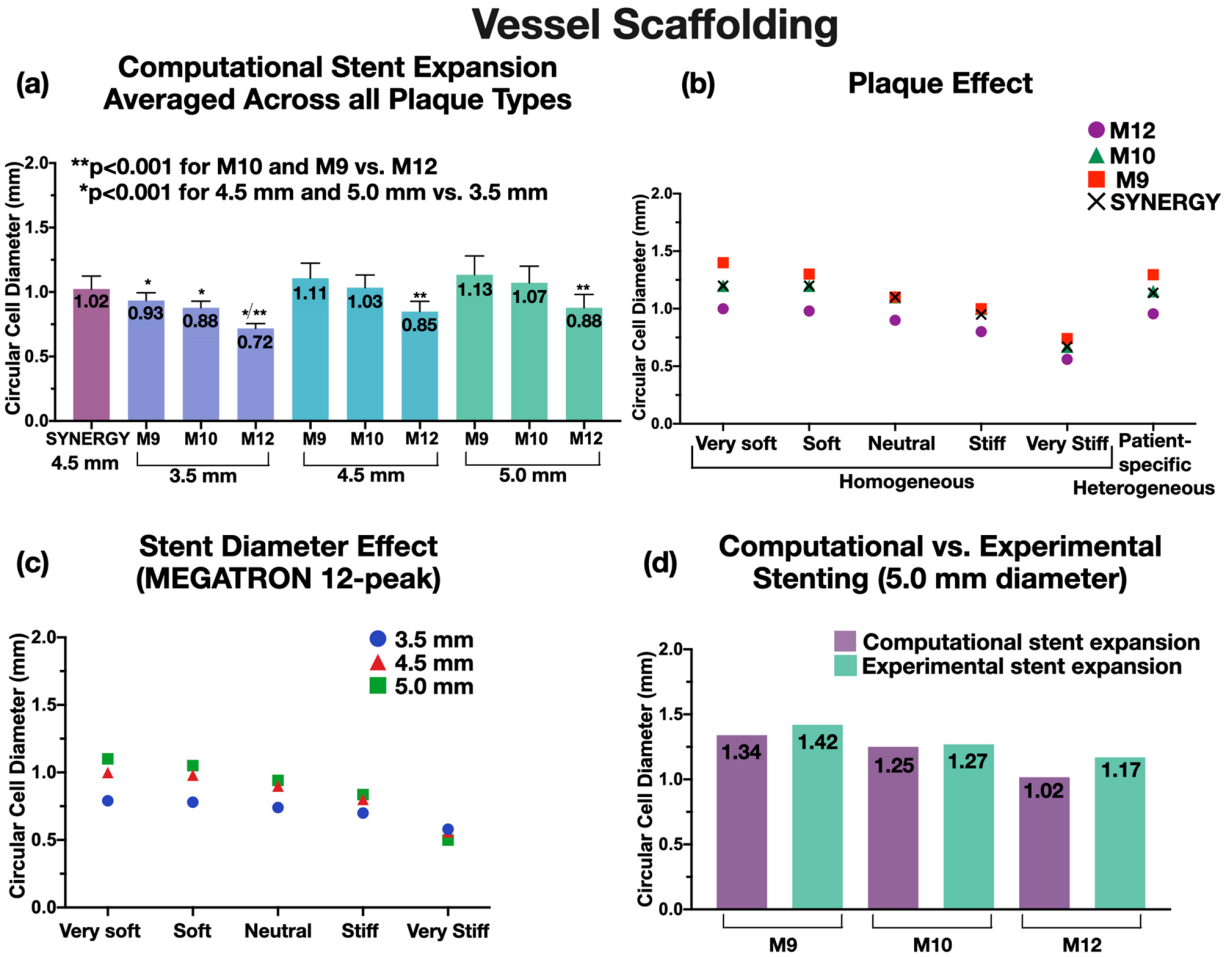
Vessel scaffolding (computational vs. experimental). Vessel scaffolding was expressed by circular cell diameter (CCD). (**a**) CCD across different stent designs and plaque types revealed that 12-peak MEGATRON had significantly better scaffolding than the other stent designs, (**b**) 12-peak MEGATRON expanded to 4.5 mm across different homogeneous and heterogeneous plaque material had smaller CCD (better scaffolding) compared to the other stent designs, (**c**) CCD of 12-peak MEGATRON across different stent diameters and plaque materials revealed that smaller diameter stents had better scaffolding than larger stents, (**d**) Computational and experimental CCD was comparable across all MEGATRON stent designs; M12: MEGATRON 12-peak, M10: MEGATRON 10-peak, M9: MEGATRON 9-peak.

**Figure 7:**
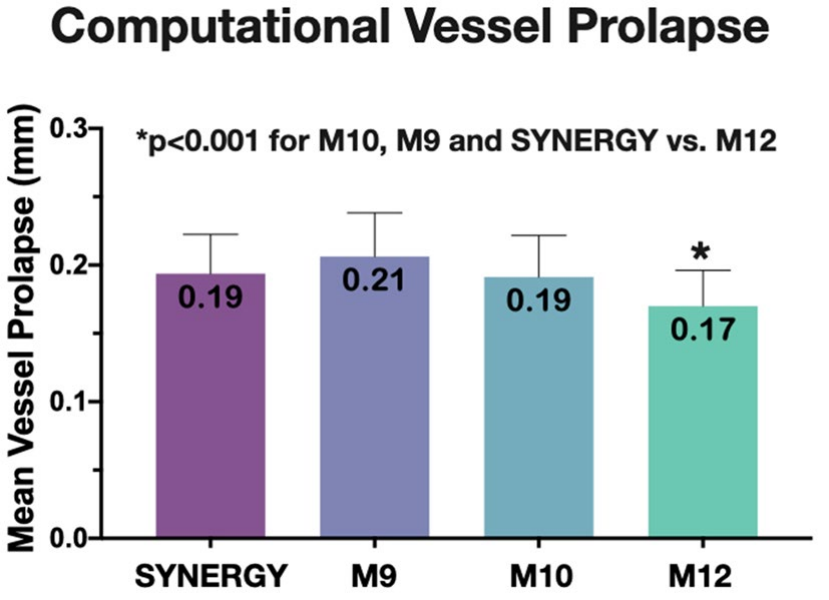
Computational vessel prolapse. Vessel prolapse across different stent designs decreased significantly with increased number of peaks (12-peak < 10-peak < 9-peak). Vessel prolapse for SYNERGY is similar to M10; M12: MEGATRON 12-peak, M10: MEGATRON 10-peak, M9: MEGATRON 9-peak.

**Figure 8:**
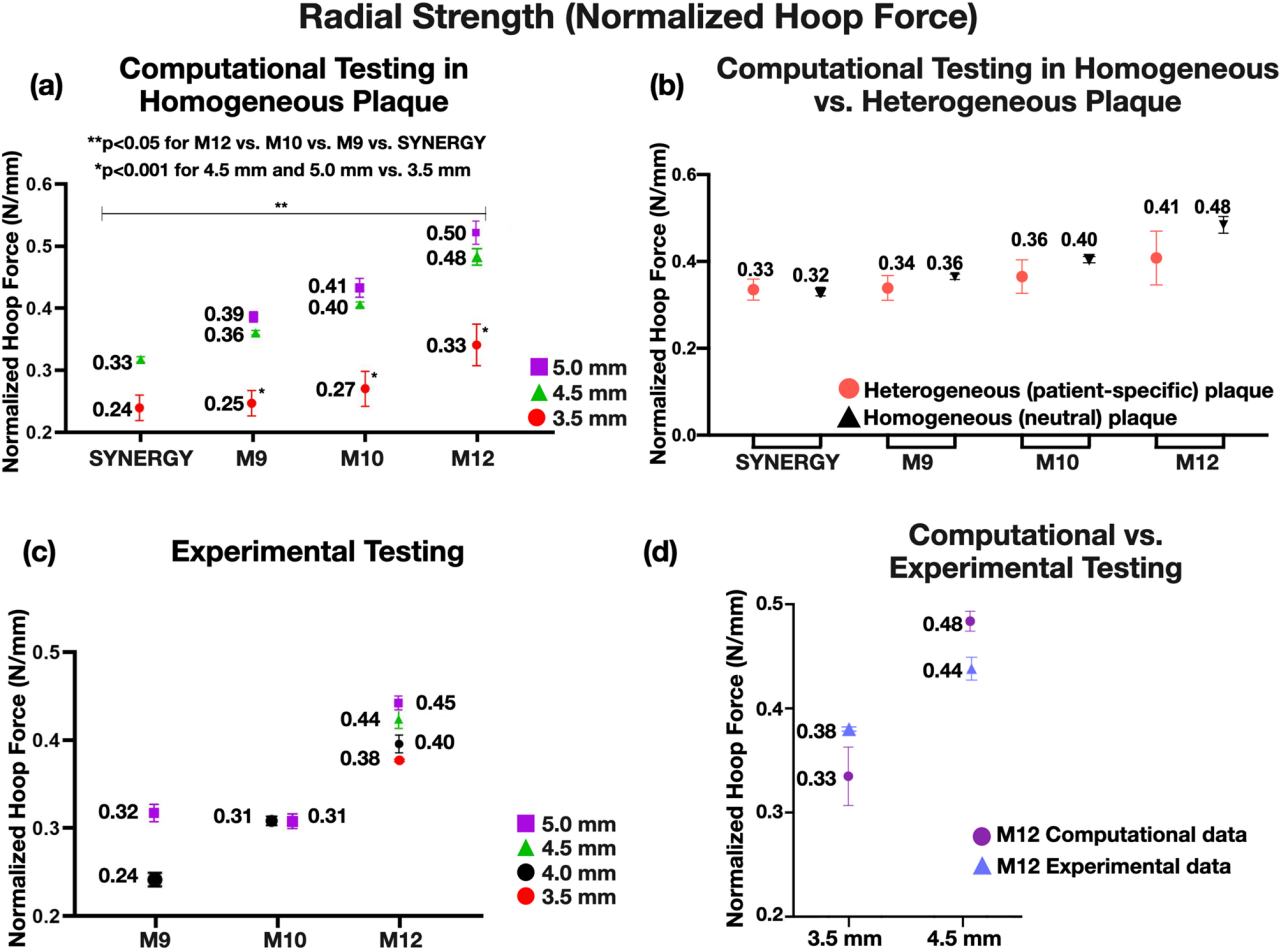
Normalized hoop force of MEGATRON and SYNERGY stent designs expanded at different diameters. (**a**) Normalized hoop force calculated computationally across different stent designs and expansion diameters for homogeneous plaque material, (**b**) Normalized hoop force calculated computationally across different stent designs for heterogeneous plaque material, (**c**) Normalized hoop force calculated experimentally across different stent designs and expansion diameters, (**d**) Computational vs. experimental normalized hoop force of 12-peak MEGATRON; M12: MEGATRON 12-peak, M10: MEGATRON 10-peak, M9: MEGATRON 9-peak.

The results of computational stent expansions were analyzed and averaged across n = 4 different patient-specific LM bifurcation geometries for all the n = 5 comparison parameters, as applied in Figs. 5, 6, 7 and 8 and Supplemental Figs. 3, 4 and 5.

### Computational stent expansion

The computational stent simulations showed that there was a dose–response relationship between the number of stent peaks and stent expansion against homogeneous soft, neutral, stiff plaques, and patient-specific (heterogeneous) plaque, in that the higher the number of peaks the higher the MSD (Fig. 5a–d). This effect was consistent across all stent expansion diameters i.e. 3.5 mm (Fig. 5a), 4.5 mm (Fig. 5b), and 5.0 mm (Fig. 5c). SYNERGY expansion pattern was comparable to 9-peak MEGATRON (Fig. 5b,d). Table 3 shows the percentage difference in stent expansion from 9-peak to 12-peak MEGATRON under different homogeneous plaque conditions. Of note, the difference in expansion of 12-peak vs. 10-peak vs. 9-peak MEGATRON vs. Synergy was amplified when stents were expanded against homogeneous soft or neutral plaque conditions. In contrast, stent expansion of 12-peak, 10-peak and 9-peak MEGATRON, and SYNERGY was similar in the extremes of homogeneous plaque conditions, i.e. very soft or very stiff plaque (Fig. 5a–c). Heterogeneous patient-specific plaques exhibited similar stiffness pattern to homogeneous soft plaques (Fig. 5b,d).

**Table 3:** Relative difference of MEGATRON 12-peak design versus 9-peak design across different homogeneous plaque conditions at different expansion diameters.

Plaque material	MSD percentage difference for MEGATRON stent designs (12-peak − 9-peak)/9-peak × 100%		
	3.5 mm expansion	4.5 mm expansion	5.0 mm expansion
	Mean ± SEM	Mean ± SEM	Mean ± SEM
Very soft	1.99 ± 0.10	1.76 ± 0.08	1.24 ± 0.23
Soft	3.79 ± 0.53	4.71 ± 0.54	4.06 ± 0.85
Neutral	3.54 ± 0.74	4.47 ± 0.53	3.60 ± 0.54
Stiff	4.21 ± 0.24	3.24 ± 0.60	1.83 ± 0.70
Very stiff	1.96 ± 0.18	1.51 ± 0.09	0.99 ± 0.21

### Vessel scaffolding

#### Computational analysis

Vessel scaffolding computational analysis represented by CCD showed that 12-peak MEGATRON had significantly smaller CCD (*p* < 0.001), and hence, better scaffolding than 9-peak MEGATRON, 10-peak MEGATRON, or SYNERGY across all homogeneous and heterogeneous plaque materials and expansion diameters (Fig. 6a,c,d). SYNERGY’s scaffolding performance was closer to 10-peak MEGATRON. The difference in vessel scaffolding of 12-peak vs. 10-peak vs. 9-peak MEGATRON was greatly attenuated with homogeneously stiffer plaques (Fig. 6b). With regards to stent expansion diameters, stents expanded to 5.0 mm or 4.5 mm had significantly larger CCD than 3.5 mm stents (*p* < 0.001) across all stent designs, and this difference was also attenuated with homogeneous stiffer plaques (Fig. 6c). Overall, these results show that stents expanding against stiffer plaques achieve lumen scaffolding in the expense of suboptimal expansion (“Computational stent expansion” section). Heterogeneous patient-specific plaques were similar to homogeneous soft plaque in terms of vascular scaffolding (Fig. 6b).

#### Experimental analysis

When all n = 3 MEGATRON designs were expanded to 5.0 mm, the 12-peak stent showed lower CCD, i.e. better scaffolding, than the 10-peak or 9-peak stent (Fig. 6d). To assess the effect of stent diameter on CCD, the 12-peak MEGATRON was expanded at different diameters (3.5 mm, 4.0 mm, 4.5 mm, and 5.0 mm). This analysis showed that the smaller the stent expansion diameter the greater the scaffolding (data not shown).

#### Computational vs. bench analysis

In bench testing of vessel scaffolding, stent expansions were carried against soft hydrogel material. To make the computational testing comparable to the experimental testing, the stents were computationally expanded against homogeneous very soft, soft and neutral plaque conditions, which were closer in terms of material stiffness to hydrogel material. Experimentally calculated scaffolding of 5.0 mm stents (of all designs) was in good agreement with the scaffolding of computationally deployed stents, yielding a difference of 6–7% (Fig. 6d).

### Vessel prolapse

#### Computational analysis

Computational simulations showed that there was an inverse dose–response relationship between stent design and vessel prolapse, the greater the number of peaks, the smaller the vessel prolapse (*p* < 0.001). Hence, greater metal coverage due to higher number of peak-to-valleys results in smaller vessel protrusion (Fig. 7). SYNERGY’s vessel prolapse performance was closer to 10-peak MEGATRON. Different expansion diameters (3.5 mm vs. 4.5 mm) did not have any significant effect on vessel prolapse (data not shown).

#### Relationship between computationally calculated vessel prolapse and scaffolding

Computational testing showed that stents with higher peaks (12-peak MEGATRON) had better vessel scaffolding and minimal prolapse compared to 10-peak or 9-peak stents. Similarly, smaller stent diameters had better scaffolding and minimal prolapse (Supplemental Fig. 3).

### Stent artery ratio (SAR)

The SAR measured as the ratio of computationally expanded stent strut area to total stented lumen area with homogeneous neutral (Supplemental Fig. 4a) and heterogeneous plaque (Supplemental Fig. 4b) conditions was greater for 12-peak MEGATRON than 10-peak or 9-peak MEGATRON. With regards to the stent size, there was a significantly inverse relationship between stent expansion diameter and SAR, in that the greater the stent diameter the smaller the SAR (*p* < 0.001; Supplemental Fig. 4a).

### Normalized hoop force

#### Computational analysis

Normalized hoop force of stents (used as an indirect measure of radial strength) computationally expanded under homogeneous neutral (Fig. 8a) and heterogeneous (Fig. 8b) plaque conditions was significantly greater with 12-peak MEGATRON than 10-peak, 9-peak MEGATRON, or SYNERGY (*p* < 0.05). Normalized hoop force of SYNERGY was similar to that of 9-peak MEGATRON. Stents expanded to 5.0 mm or 4.5 mm diameters hoop force was significantly higher than 3.5 mm stents. This effect was consistent across all stent designs (*p* < 0.001, Fig. 8a).

In heterogeneous plaque, directly measured radial strength was slightly greater for the 12-peak MEGATRON than 10-peak, 9-peak MEGATRON, and SYNERGY for all n = 4 LM geometries (Supplemental Fig. 5).

#### Experimental analysis

In bench testing, the 12-peak MEGATRON showed greater hoop force than 10-peak or 9-peak stent designs, irrespective of stent expansion diameter. When compared across different expansion diameters, 5.0 mm stent expansions consistently showed greater normalized hoop force value than the smaller stents across all stent designs (Fig. 8c).

#### Computational vs. experimental analysis

Stent hoop force by computational and experimental testing was comparable (an approximate difference of 10%; Fig. 8d), suggesting that the computational approach can reliably replicate the experimental conditions.

## Discussion

In this work, we performed independent computational and experimental testing of different designs of a novel everolimus-eluting stent (i.e. MEGATRON). MEGATRON is purpose-built for LM and large-sized coronary artery interventions. Our computational study is an extension of the previous experimental optimization work, within the context of the design process of MEGATRON stents. The comparative MEGATRON stent designs had discrete geometrical differences with 9 peaks, 10 peaks, and 12 peaks in every crown, with the 12-peak being the final MEGATRON commercial design. The three MEGATRON designs were compared computationally and experimentally with each other, as well as against the existing SYNERGY. The mechanical performance of these stent designs was tested computationally under various plaque material properties using patient-specific LM bifurcation anatomies of varying disease burden and complexity. The mechanical performance of the stents was also tested in benchtop setups that were blind to the computational results. Our study showed that: (i) The 12-peak MEGATRON had greater stent expansion, vessel scaffolding, normalized hoop force/radial strength, and stent-to-artery ratio, as well as lesser vessel prolapse than the 10-peak and 9-peak designs, (ii) The mechanical performance of the commercial SYNERGY design was in between 9-peak and 10-peak MEGATRON, (iii) With regards to different stent expansion diameters, smaller stent sizes had better stent-to-artery ratio and vessel scaffolding. Normalized hoop force was greater with larger stent diameters, whereas stent diameter did not have any significant impact on vessel prolapse, (iv) Computational testing revealed very comparable stent performance data compared to bench testing, speaking to the robustness of the computational simulations to study stent designs under different patient-specific plaque conditions.

There are several novelties within our work: (i) Use of a novel stent platform i.e. MEGATRON, (ii) Testing of stent performance (multifactorial systemic analysis) across a wide spectrum of stent designs, stent sizes and plaque stiffness using a patient-specific computational simulations platform, (iii) Computational testing of stent performance in patient-specific LM bifurcations that incorporates realistic coronary anatomies (including lumen and wall), (iv) Incorporation of OCT based patient-specific longitudinal and circumferential plaque heterogeneity, (v) Blind head-to-head comparison of computational vs. experimental stenting.

In order to achieve favorable stenting outcomes, particularly at the LM bifurcations, the stent design should conform to the vessel curvature and bifurcation with uniform and adequate scaffolding, minimal prolapse, and optimal expansion^36^. The patient-specific computational stent testing in this study, showed that the greater the number of peaks in each stent segment, the better the mechanical stent performance (i.e. expansion, scaffolding, and prolapse). One could hypothesize that increasing the number of peaks could have better expansion, scaffolding and negligible prolapse, however, there would be a significant trade-off with increased stent profile, reduced stent deliverability and potentially higher rates of in-stent restenosis^7,37^. For that reason, 12-peak stent design was considered optimal and became the commercially available version of the stent. Improved stent scaffolding and hoop force/radial strength play fundamental role in overall stent performance and subsequent clinical outcomes, and this becomes more pertinent to LM stenting which requires stronger metallic scaffolds to withstand the radial wall resistance to stent expansion. Furthermore, our computational study revealed important findings related to the role of plaque material properties in stent performance. In particular, the positive effect of stent design and expansion diameter on tissue scaffolding was more prominent with softer plaques and minimal in stiffer plaque conditions. We found that the SAR, reflecting the metal coverage of the lumen, was higher with MEGATRON 12-peaks compared to other stent designs. Higher SAR, has been associated with in-stent restenosis and stent thrombosis^7^. However, a recent study showed that SAR has no significant impact on LM stenting outcomes, particularly in larger stent sizes (> 3.5 mm)^38^.

Another major finding in this study is that the computational testing showed similar results compared to experimental in terms of vessel scaffolding and hoop force across different stent designs and sizes. Computational stent testing was carried out on a well-founded and validated simulation platform^28^. To date, various stenting simulation studies have experimentally validated the mechanical stent performance, such as expansion, fatigue/fracture, strength and flexibility^12,13,39^, as well as predicted stent and lumen stresses to assess potential in-stent restenosis^40^. But none of these stenting simulations was done under realistic patient-specific plaque conditions. Our study highlights the robustness and ability of our computational stenting platform to faithfully replicate the realistic stent behavior under patient-specific conditions that cannot be studied adequately in bench or animal studies. Computational simulation testing of stents enables us to study how various stent designs interact with different plaque materials in patient-relevant vessel environments. Computational stenting simulations can be applied in virtual (in-silico) clinical trials using real patient data and surrogate (acute) endpoints (e.g. stent expansion, apposition, vessel scaffolding, side branch jailing, fluid dynamics), highly predictive of long term clinical outcomes. These virtual clinical trials can guide the design of actual clinical trials with real patients and clinical outcomes, thereby saving time and resources. Conceivably, these advantages of computational stenting simulations might open a whole new perspective in stent industry, making the stent testing, development and regulatory approval processes faster and more cost-effective.

There were several limitations within this work: First, we studied a limited number (n = 4) of LM bifurcations. However, the results were very consistent across all n = 4 cases, speaking to the robustness of our results. Furthermore, we deployed a multifactorial analysis of 4 (stent designs) by 3 (stent diameters) by 6 (plaque materials), in all n = 4 LM bifurcations, resulting in a total of n = 232 computational simulations. Future larger virtual clinical studies are warranted to confirm the important findings of this exploratory computational study. In this work, we did not perform analysis of local fluid dynamics and structural integrity of stents after maximum expansion. This biomechanical analysis is subject to our ongoing research. In our study, the focus was on the mechanical performance of the stents and we did not factor the biological response of the arterial wall to stent expansion. However, extensive studies have shown that mechanical stent performance is likely the dominant determinant of clinical outcomes post-stenting^41^.

In conclusion, this is the first study to test the mechanical performance of a new stent (i.e. MEGATRON 12-peak) purposefully purposely built for LM and large proximal coronary vessels, using a well-validated patient-specific computational simulation and experimental setup. The study provided consistent data showing that 12-peak MEGATRON has better performance than the 9-peak, 10-peak MEGATRON versions or SYNERGY. This study opens new perspectives, introducing a time- and cost-effective strategy for stent research and development. Further virtual clinical trials to confirm the study findings in a larger data set are warranted.

## Supplementary Information

Supplementary Information.

## Abbreviations

3D: Three-dimensional
CCD: Circular cell diameter
DES: Drug-eluting stent
FEA: Finite element analysis
LM: Left main
LAD: Left anterior descending artery
LCX: Left circumflex artery
MSD: Mean stent diameter
OCT: Optical coherence tomography
SAR: Stent artery ratio
