# Supplementary material for: Computational and experimental mechanical performance of a new everolimus-eluting stent purpose-built for left main interventions
Source: Sci Rep. 2021 Apr 22;11:8728. doi: 10.1038/s41598-021-87908-2 (PMC8062511; doi:10.1038/s41598-021-87908-2)
Supplement: Supplementary file 1 — Supplementary Information. [file 41598_2021_87908_MOESM1_ESM.docx]

**Computational and Experimental Mechanical Performance of a**

**New Everolimus-eluting Stent Purpose-built for Left Main Interventions**

Saurabhi Samant, MBBS^1*^; Wei Wu, PhD^1*^; Shijia Zhao, PhD^1^; Behram Khan, MD^1^;

Mohammadali Sharzehee, PhD^1^; Anastasios Panagopoulos, MD^1^; Janaki Makadia, MBBS^1^; Timothy Mickley, BSME^2^; Andrew Bicek, PhD^2^; Dennis Boismier, MSME^2^;

Yoshinobu Murasato, MD, PhD^3^; Yiannis S. Chatzizisis, MD, PhD^1^

^1^Cardiovascular Division, University of Nebraska Medical Center, Omaha, NE, USA

^2^Boston Scientific, Maple Grove, MN, USA

^3^Department of Cardiology, National Hospital Organization, Kyushu Medical Center, Fukuoka, Japan

*The first two authors contributed equally to the manuscript

**Word count:** 4,364

**Funding source:** National Institutes of Health (R01 HL144690), Boston Scientific Inc., Dr Vincent Miscia Cardiovascular Research Fund

**Disclosures:** Yiannis S. Chatzizisis has received speaker honoraria, advisory board fees and a research grant from Boston Scientific Inc. and a research grant from Medtronic Inc. Dr Chatzizisis has domestic and international patents pending entitled “Computational simulation platform for the planning of interventional procedures” (U.S. Patent Application No. 17/076,213 and International Application No. PCT/US2020/057304). Timothy Mickley, Andrew Bicek, Dennis Boismier are employees of Boston Scientific Inc. All other authors have nothing to disclose.

**Corresponding author:**

*Yiannis S. Chatzizisis, MD, PhD*

Cardiovascular Biology and Biomechanics Laboratory

Cardiovascular Division,

University of Nebraska Medical Center

982265 Nebraska Medical Center, Omaha, NE, 68198 USA

Tel: (402) 559-5156

Fax: (402) 559-8355

E-mail: [ychatzizisis@icloud.com](mailto:ychatzizisis@icloud.com)

**Supplemental Material**

**Supplemental Figures**

**Supplemental Figure 1. Computational analysis of hoop force. (a)** Computationally simulated radial crimping of MEGATRON 12-peak expanded to 4.5 mm, (**b)** Comparison between computationally and experimentally calculated hoop force showing high agreement of maximum hoop force.

**
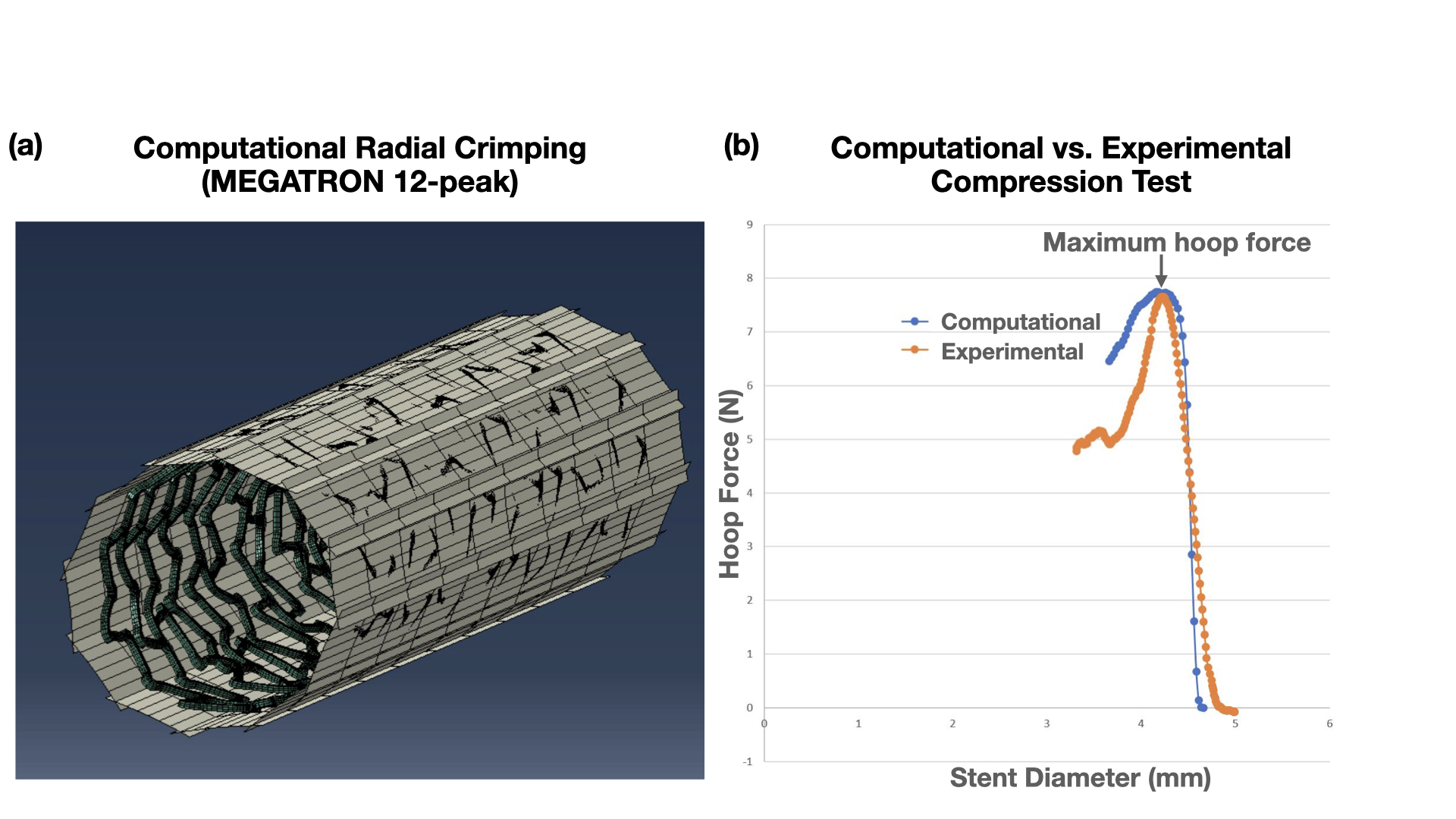
**

**Supplemental Figure 2. Experimental setup for the calculation of hoop force.** Compression tester (MSI RX-Series Radial Expansion Force Gage) showing the 12-wedge shaped force elements that rotate freely and touch the tested stent (specimen inserted in head) in the center. The radial force applied by the stent was measured and hoop force was then calculated as radial force/2π. In the graph, the distance between the first dot from right (red solid line, maximum expansion) to the third dot (orange dotted line) corresponds to 15% reduction in stent diameter.


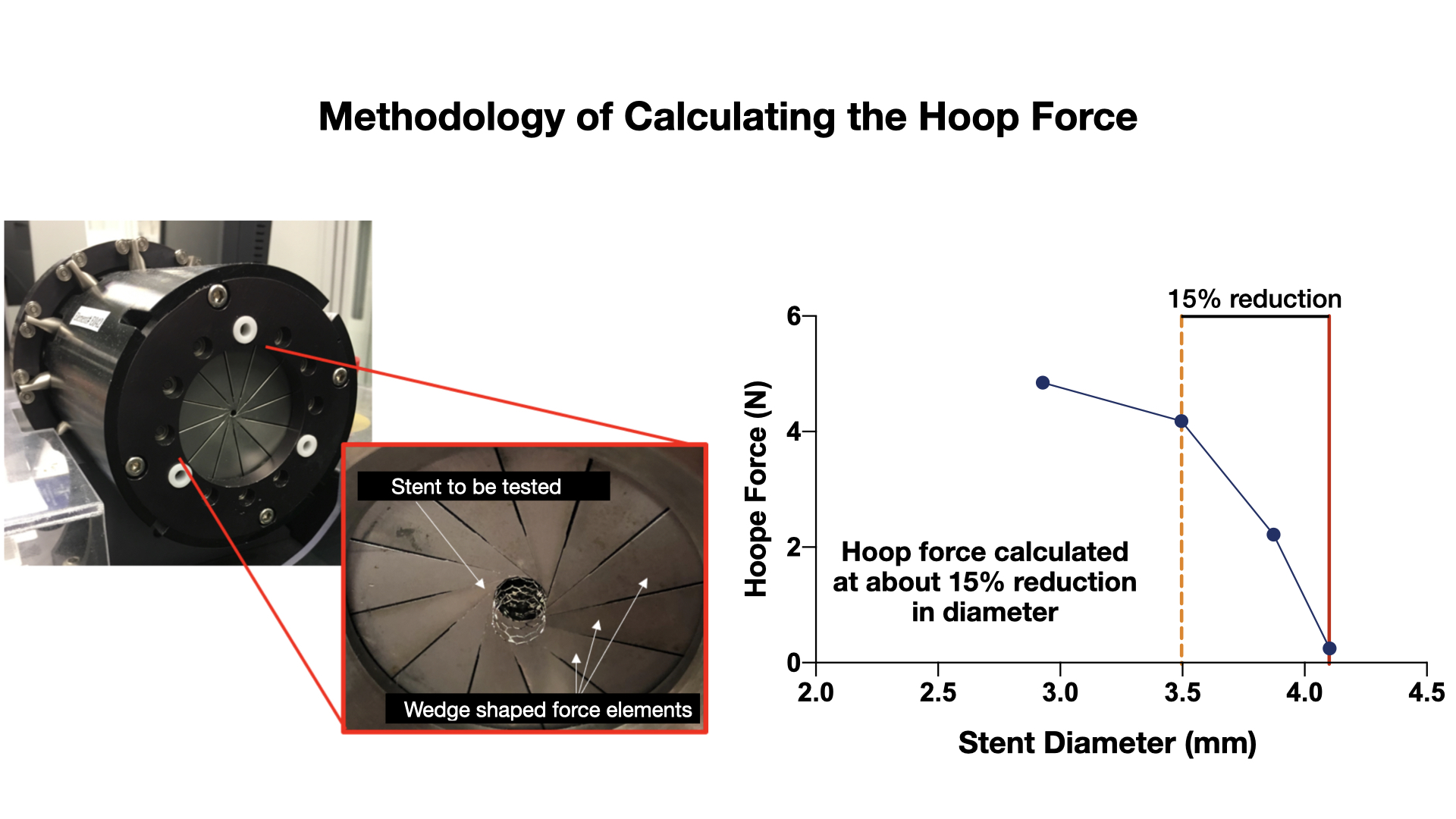


**Supplemental Figure 3. Vessel prolapse vs. vessel scaffolding.** Inverse relationship between vessel prolapse and vessel scaffolding shown by computational testing of different stent designs.


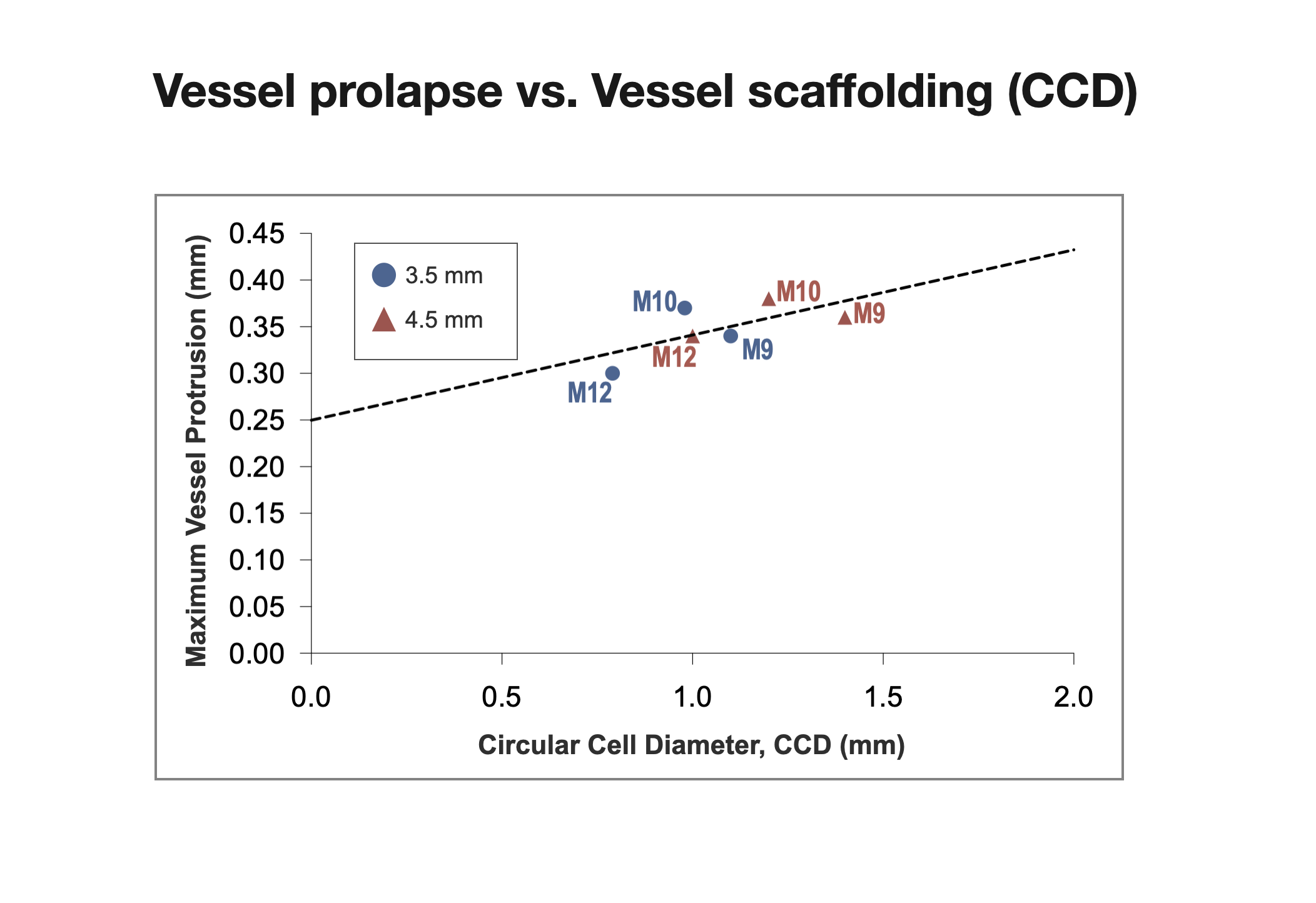


**Supplemental Figure 4. Computational stent-to-artery ratio. (a)**The stent-to-artery ratio was calculated across different stent designs and expansion diameters under homogeneous (neutral), **(b)** and heterogeneous (patient-specific) plaque material; M12: MEGATRON 12-peak, M10: MEGATRON 10-peak, M9: MEGATRON 9-peak.


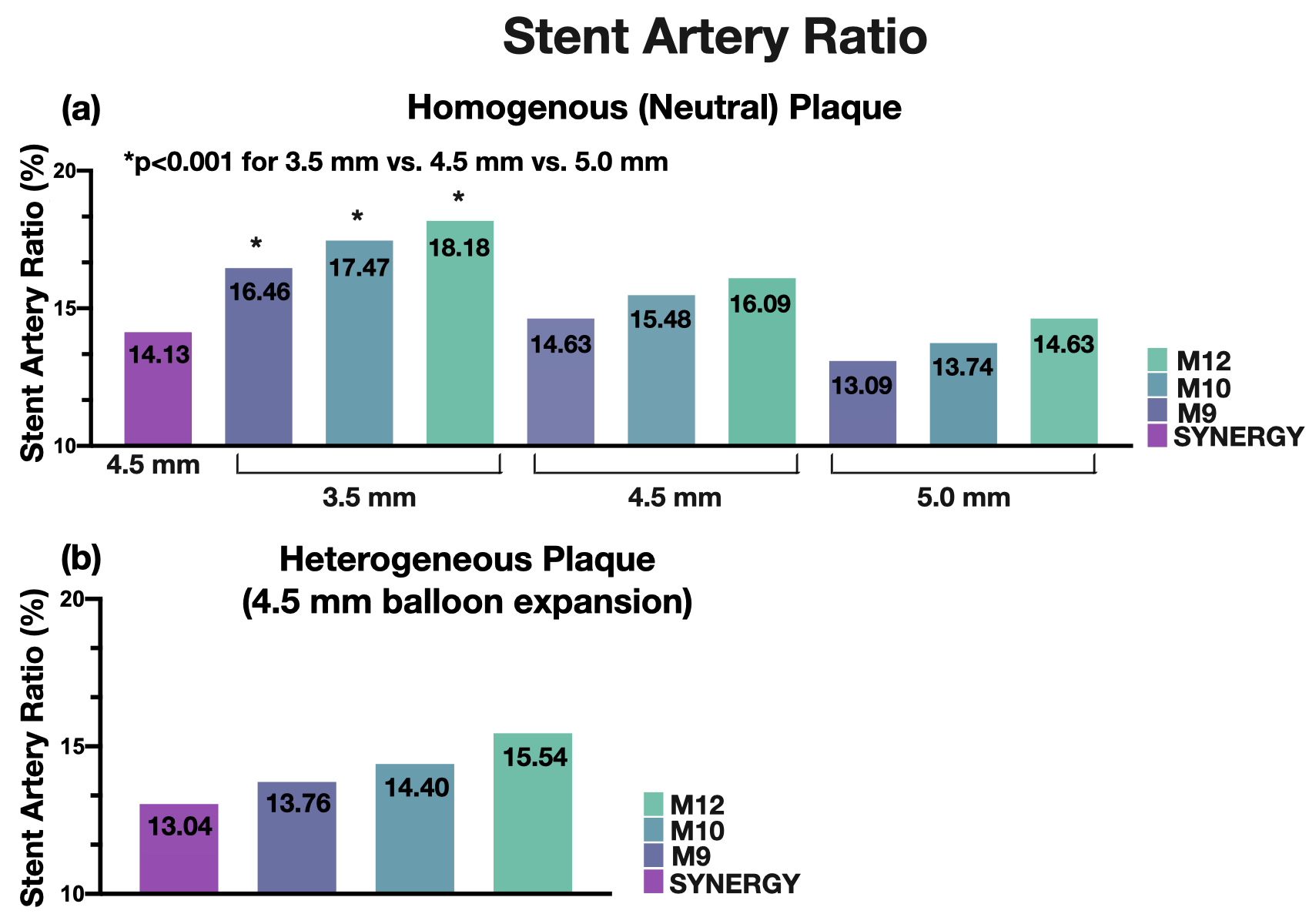


**Supplemental Figure 5.** **Computational Radial Strength.** Radial strength of different stent designs (MEGATRON 9-peak, 10-peak, 12-peak and SYNERGY) expanded at 4.5 mm stent diameter against patient-specific plaques with heterogeneous materials; M12: MEGATRON 12-peak, M10: MEGATRON 10-peak, M9: MEGATRON 9-peak.


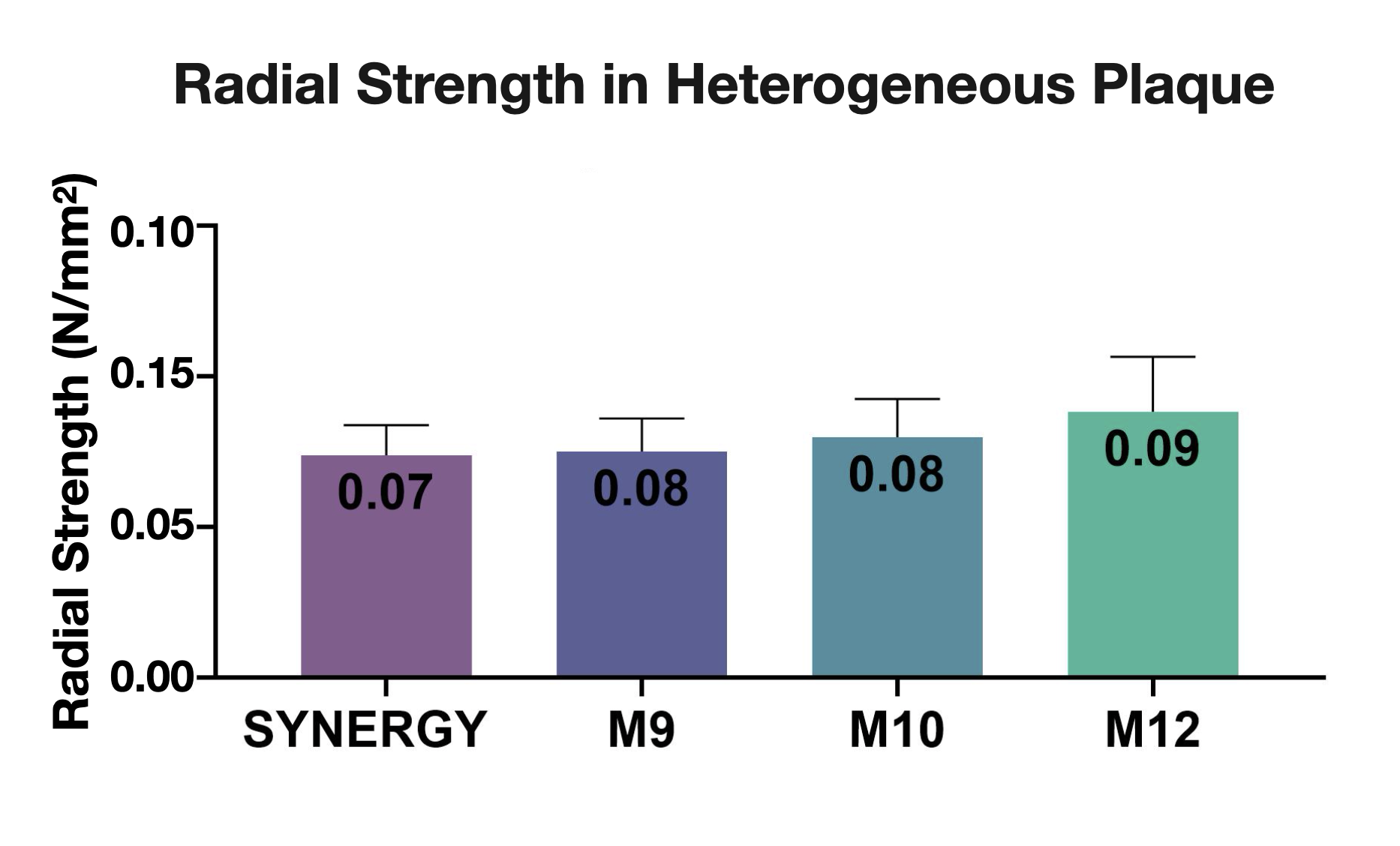


**Supplemental Tables.**

**Supplemental Table 1.** Coefficients for the material models used in computational stenting simulations for Megatron.

|  | C10 (MPa) | C20  (MPa) | C30  (MPa) | C40  (MPa) | C50  (MPa) | C60  (MPa) | Yield stress (MPa) |
| --- | --- | --- | --- | --- | --- | --- | --- |
| **Normal wall** | 6.52e-3 | 4.89e-2 | 9.26e-3 | 0.76 | -0.43 | 8.69e-2 | - |
| **Very soft** | 0.045 | 0.17 | -0.13 | 0.11 | - | - | 0.19 |
| **Soft** | 0.01 | 0.49 | 4.13 | - | - | - | 2.07 |
| **Neutral** | 0.06 | 4.28 | -21.36 | 69.36 | - | - | 6.02 |
| **Stiff** | 0.11 | 9.06 | - | - | - | - | 8.35 |
| **Very stiff** | 0.21 | 64.86 | -3.5e3 | 1.999e5 | - | - | 2.87e4 |
|  | Elastic Modulus  (GPa) | 0.2% Yield strength  (MPa) | Tensile Strength MPa) | Elongation  (%) |  |  |  |
| **Pt-Cr** | 203 | 480 | 834 | 45 |  |  |  |
